# Applied Mindfulness for Physician Wellbeing: A Prospective Qualitative Study Protocol

**DOI:** 10.3389/fpubh.2022.807792

**Published:** 2022-02-11

**Authors:** Elli Weisbaum, Nicholas Chadi

**Affiliations:** ^1^Institute of Medical Science, Faculty of Medicine, University of Toronto, Toronto, ON, Canada; ^2^Buddhism, Psychology and Mental Health Program, New College, Faculty of Arts and Sciences, University of Toronto, Toronto, ON, Canada; ^3^The Hospital for Sick Children, Toronto, ON, Canada; ^4^Department of Pediatrics, University of Montreal, Montreal, QC, Canada; ^5^Division of Adolescent Medicine, Sainte-Justine University Hospital Centre, Montreal, QC, Canada

**Keywords:** mindfulness, wellbeing, burnout, physicians, qualitative, protocol, intervention, applied

## Abstract

**Background:**

Physician burnout has significant adverse impacts on the wellbeing of individual physicians, and by extension the healthcare delivery systems of which they are part. Mindfulness is consistently cited as a pragmatic approach to effectively address burnout and enhance physician wellbeing. However, very few empirical studies have been published on Mindfulness Based Interventions (MBIs) for physicians. Moreover, the majority of these studies have been quantitative, leaving a gap in understanding the practical application of mindfulness in the context of physicians' daily lives.

**Objectives:**

This paper outlines the rationale, development and design of a novel prospective qualitative study examining the acceptability, feasibility, and pragmatic application of a mindfulness intervention for physician wellness.

**Methods:**

The study will be conducted in three groups of at least 8 practicing physicians from a broad range of medical specialties at a tertiary care hospital in a large urban center in Eastern Canada. The intervention will consist of an innovative program based on the teachings of internationally renowned scholar and Zen Master Thích Nhãt Hạnh. It will include 5 weekly 2-h mindfulness sessions delivered by two health providers trained in mindfulness and in the teachings of Thich Nhat Hanh. The primary outcome measure will be an in-depth Thematic Analysis of post-program semi-structured interviews. Field data will also be collected through participant observation. The study will be theoretically grounded within the interpretive paradigm utilizing “the Mechanisms of Mindfulness Theory”. An external advisory committee formed by four senior members of Thích Nhãt Hạnh's community will provide guidance across all phases of the study.

**Discussion:**

Our innovative approach provides a new framework to further understand the mechanisms by which mindfulness interventions can impact physician wellbeing, and by extension their patients, colleagues, and broader healthcare systems. Through a clear articulation of the rigorous application of accepted procedures and standards used in our protocol, this paper seeks to provide a roadmap for other researchers who wish to develop further studies in this area. Lessons learned in the preparation and conduction of this study can be applied to other healthcare contexts including non-physician health provider wellbeing, clinical care, and population-level mental health.

## Introduction

Physician wellness can positively impact the quality of patient care, frequency of medical errors, patient satisfaction and clinician professionalism (1–7). Calls have been made across the literature, and from international medical associations, for further research to be conducted on pragmatic approaches to effectively address physician burnout and enhance physician wellbeing (1, 4, 8–11). Prevalence of physician burnout is characterized as nearing or exceeding 50% of training and practicing physicians (4, 10, 12). Risk factors related to physician burnout are difficult to address, as they are situated at a complex intersection of the occupational environment, individual behaviors and the broader culture of medicine (3).

Physician burnout can lead to absenteeism, tardiness, reduced job commitment and increased physician turnover (5, 13, 14). The costs associated with replacing a single physician have been estimated at two to three times a physician's annual salary (15). Conversely, moderate investments spent on wellness programs have can directly reduce costs associated with medical leave and absenteeism (16, 17). Across the literature, it is recognized that the burden of addressing burnout should be a shared endeavor between individual physicians and the wider healthcare system. Therefore, an integrative and collaborative effort that brings together multiple modalities—including interventions, trainings, research and resources—to create internal and external change at the individual and systemic levels is necessary to address this global epidemic within healthcare (8, 15, 16, 18).

Mindfulness-based interventions are consistently recommended across the literature as a promising approach for addressing physician burnout and enhancing physician wellbeing (15, 18). Mindfulness is described by acclaimed scholar and Zen master Thích Nhãt Hạnh as the observational awareness of what is happening inside and around oneself in the present moment (e.g., awareness of relevant stimuli's impacts on one's internal/external states) (19, 20). Mindfulness can be experienced alone or with others, through both formal and integrated practice (21). Therefore, mindfulness lends itself well to a multi-session group intervention format during which participants can develop foundational mindfulness skills, which they can further enhance with individual practice between sessions.

Very few studies have been conducted that bring together mindfulness and physician wellbeing. In the broader literature, mindfulness has been shown to have a wide range of potential health benefits, including: reduced anxiety and stress, increased wellbeing, self-regulation, emotional-regulation, motor skills and empathy, along with greater connectivity between brain regions associated with prosocial behaviors (21–30). The small number of initial empirical studies that have been conducted with physicians show a promising potential for mindfulness to positively benefit physician wellbeing, decrease emotional exhaustion and perceived stress, increase compassion/empathy and enhance patient-centered care (31–38). These studies have shown that MBIs have the potential to impact physicians across burnout as measured by the Maslach Burnout Inventory (e.g., emotional exhaustion, depersonalization, and personal accomplishment) (33, 34, 36–39). Not all studies report statistically significant improvements. For example, a study which applied a Mindfulness-Based Stress Reduction intervention with medical, surgical and primary care residents at Radboud University in the Netherlands Verweij et al. (40) found no significant difference in emotional exhaustion between the active and control groups. However, significantly greater improvements were reported by the intervention group than the control group in relation to personal accomplishment, self-compassion and perspective taking (40). The majority of these preliminary studies with physicians have been quantitative, leaving a gap in the understanding of the lived experience and practical application of mindfulness by physicians in the context of their daily lives.

This study will apply a qualitative research framework to support the study's central aim to understand the acceptability, feasibility, and pragmatic application of mindfulness in the context of physicians' daily lives. Qualitative methods are particularly well-suited for studying and understanding human behavior in the context of the social world (41, 42). A distinguishing feature of qualitative research is its use of a research question, rather than hypotheses (42, 43). This is particularly relevant to our study, as human behavior and interactions are often more complex than what can be preemptively or objectively defined or controlled (44).

## Methods and Analysis

### Study Design

This is a prospective qualitative study of physician wellbeing that will be conducted during and following the completion of a 5-week Applied Mindfulness training program. Through the rigorous application of high-academic research standards for qualitative healthcare research, this study aims to contribute new knowledge to prevent physician burnout by bringing together the following three pillars: (1) Physician wellbeing; (2) mindfulness; and (3) qualitative research.

### Research Question

The research question for this study, along with two guiding sub questions to help orient the process of analysis, are as follows:

Research Question: How do physicians experience, make sense of, and engage with a 5 week Applied Mindfulness program and what is the impact of the program on their personal wellbeing in the context of their daily lives?

Guiding sub-questions/further areas of inquiry: How, where and in what context(s), do participants apply mindfulness? How does mindfulness impact participants' perceived *relationship* to themselves, colleagues, patients and the context in which they work?

### Population

#### Inclusion and Exclusion Criteria

The study will recruit physicians in active medical practice within the Greater Toronto Area (GTA), Canada. Medical students and other health care personnel (HCPs) will be excluded. Studies on MBIs conducted with physicians, medical students and HCPs show that, while there are some similarities across their experiences, there are also several areas of significant differences (e.g., how mindfulness impacts studying for exams, and different sets of power dynamics) (45–49). Therefore, based on this study's research question the sample will be limited to physicians, fellows and residents all of whom will have completed undergraduate medical training at the time of enrollment (see Table 1 for inclusion/exclusion criteria).

**Table 1:** Inclusion and exclusion criteria.

**Inclusion criteria**	**Exclusion criteria**
• Able to independently consent to study • Physician, fellow or resident practicing medicine within the GTA • Self-reported good standing with the College of Physicians and Surgeons of Ontario or College of Family Physicians of Canada • Fluent in English • Able to attend five 2-h sessions, either at midday or evening, over 5 consecutive weeks • Persons of all faiths and genders	• Planning to participate in another mindfulness training program during the delivery of the AMP-MP • Unable to adhere to study procedures

#### Sampling

The minimum sample size for this study (three groups of eight participants) is based on the theory of information power, which establishes that sample size for a qualitative study can be based on the amount of information a sample holds in relation to the aims of the study, rather than being based on a formula or perceived redundancy (50). According to Malterud et al. (50), five factors have an impact on information power. These include: (1) study aim; (2) sample specificity; (3) use of established theory; (4) quality of dialogue; and (5) analysis strategy. The greater the information power of a single sample, the smaller the sample size needs to be. Based on the inclusion/exclusion criteria (outlined above) and purposive sampling approach (described below) the samples in this study are considered to have high information power. This is due to the focused and narrow aim, participants that share the highly specific characteristic of being active physicians, the use of established qualitative theory and precise approach to the dialogue between researcher and participant (50).

#### Purposive Sampling Approach

A purposive sampling approach will be applied to enhance the sample specificity and information power. Purposive sampling allows the researcher to increase the sample specificity by selecting participants who hold specific characteristics (e.g., experience and knowledge) that are particularly relevant to the study aims, as guided by the research question (50). Participants in this study will be purposefully selected for the characteristics of (1) longer years in medical practice (providing greater knowledge of the lived experience of being a physician); and (2) fewer years of mindfulness practice (bringing a more novice perspective of mindfulness experience). In order to apply this purposive sampling approach, a question regarding number of years in medical practice and experience with mindfulness will be included in the initial online recruitment and registration survey. Participants will be accepted into the study with priority given to those who report longer years practicing medicine and fewer years of experience with mindfulness. Our sample will be drawn from physicians who practice in the Greater Toronto Area (GTA) which represents one of the most diverse workforces in Canada.

## Procedure

### Recruitment and Enrollment

Participants will be recruited through a combination of electronic email, poster and in-person announcements. Electronic mail-outs will be sent through hospital leadership and university channels. Responses will be screened for inclusion/exclusion criteria and the purposive sampling approach will be applied to prioritize the order of enrolment.

### Mindfulness Intervention Content

The intervention is called the Applied Mindfulness Program (AMP) for physicians. The mindfulness content to be delivered within this study represents an underexplored area within the field of MBIs. It is based on the teachings of internationally renowned scholar, Zen Master and Noble Peace Prize Nominee Thích Nhãt Hạnh (see Table 2 for overview of the program's weekly themes and content). While his work is internationally recognized as seminal, to date no MBIs have been based explicitly on his teachings. Thích Nhãt Hạnh's work was selected as the basis for this research study's mindfulness program because of several key characteristics. These include his work's focus on: (1) the *pragmatic application* of mindfulness into daily life to underpin the cultivation of personal wellbeing; and (2) impacting both individual and systemic change. This study's lead investigator's (EW) long-term background and training with Thích Nhãt Hạnh's community provides rich and novel access to this body of knowledge.

**Table 2:** AMP-MP weekly themes and content.

**Session**	**Theme**	**Content**
1	Orientation and foundations for practice	Admin (including consent forms) *Opening practice* Lecture: intro to mindfulness/foundations of practice (including Four Noble Truths) *Mindful eating* Practice: attention to bell/breath *Discussion* Take home activity: Awareness of breath
2	Identifying and transforming habits	*Opening practice* *Take Home Activity check-in: discuss application between sessions* Lecture: Habit energies *Mindful eating* Practice: Mindful walking activity *Discussion* Take home activity: Mindful walking
3	Nourishing our mind	*Opening practice* *Take Home Activity check-in: discuss application between sessions* Lecture: The four nutriments and store consciousness *Mindful eating* Practice: Body awareness practice *Discussion* Take home activity: Mindful eating
4	Skillful communication	*Opening practice* *Take Home Activity check-in: discuss application between sessions* Lecture: Interbeing (interconnection), Empathy fatigue vs. compassion *Mindful eating* Practice: Loving kindness/Kind intention *Discussion* Take home activity: Application in daily life project/worksheet
5	Integration and application	*Opening practice* *Take Home Activity check-in: discuss application between sessions* Lecture: Wellbeing/happiness as products of practice (Eightfold Path + Program review) *Mindful eating* Practice: tea/coffee meditation/closing ceremony (presentations of take-home application projects from Session 4) *Discussion* Closing circle/intention setting

To inform the rigorous and authentic use of Thích Nhãt Hạnh's work, an iterative reciprocal relationship was established at the outset of this research study with his international Plum Village community. This was done through the establishment of an advisory committee made up of four senior monastic teachers in the tradition of Thích Nhãt Hạnh and Plum Village. This committee will provide guidance and feedback across all phases of the study. This approach was informed by precedents from Ingenious Research Methods (51), along with calls in the literature to enhance the quality and standards of MBIs by collaborating with the traditional wisdom communities upon which their content draws (52, 53).

#### Mindfulness Intervention Delivery and Study Flow

**Figure 1:**
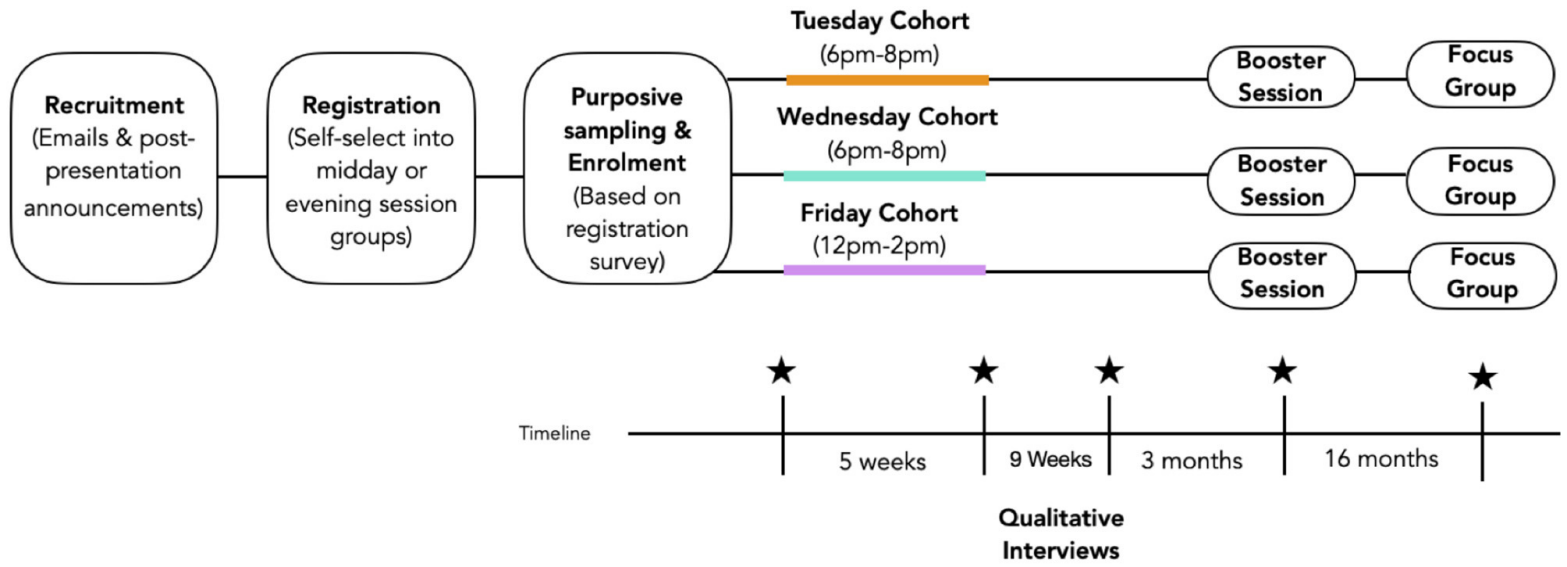
Visual representation of study flow.

Program sessions will be scheduled to take place on a weekly basis on the same time and day for each group for five consecutive weeks (2 h per session) and will have a minimum of 8 and a maximum of 23 participants in each group. Each program session consists of a mixture of didactic lectures, hands-on experiential Applied Mindfulness practices and group discussions. An overview of program structure can be found in Table 3 and a visual representation of the study flow can be found in Figure 1. Further details on the program design and content will be discussed in detail in a forthcoming paper.

**Table 3:** Overview of program structure.

Program length	1 session per week over 5 weeks
Session length	2 h (30 min added to the first session for study administration)
Booster session	1 × 2 h (3 months after program)
Focus group	1 × 1.5 h focus group (16 months after program)
In-session content/delivery	Mixture of learning styles, including didactic lectures, group discussion/sharing, hands-on practices
Additional content/delivery	Take home activities, journaling prompts and audio recordings of the in-session didactic lectures

Although the mindfulness intervention was designed to be delivered in-person, it can be adapted to a fully online setting using video-conferencing technology, should public health measures (e.g., related to COVID-19) prevent the delivery of an in-person intervention. Both authors have extensive experience delivering and studying mindfulness interventions delivered online (54–56). Emerging literature also suggests that online delivery of mindfulness-based interventions for medical professionals and trainees represents a promising mode of delivery which would benefit from further study (57, 58).

The mindfulness intervention will be delivered to three distinct groups (Table 4). The reason for having three groups is to account for physicians' complex schedules. At the time of recruitment, participants will be invited to self-select into the group that is most compatible with their schedule through the secure online REDCap platform. This will allow all participants to receive the mindfulness training by the end of the study period.

**Table 4:** Study groups.

**Day of the week**	**Time of day**
Tuesday	6:00 p.m.−8:00 p.m.
Wednesday	6:00 p.m.−8:00 p.m.
Friday	12:00 p.m.−2:00 p.m.

### Program Facilitators

The mindfulness training program will be led by two facilitators who are not part of the primary research team and have professional training in mindfulness, along with a committed personal practice rooted in the tradition of Thích Nhãt Hạnh and Plum Village. The facilitators will use a program manual as a guide for each session's structure and content. The same facilitators will facilitate all of the program sessions and a manual checklist will be used by a participant observer to track the content presented across the different sessions.

### Ethics and Participant Safety

A mindfulness-based program is not generally seen as entailing any significant risks to the physical and psychological safety of participants (59). Some participants might experience temporary discomfort or unpleasant physical, emotional, or mental experiences while exploring the topic of wellbeing either during mindfulness sessions, or during qualitative follow-up interviews. Participants will be informed that they may withdraw from the study or follow-up interviews at any time. Institutional ethics approval has been obtained from The Hospital for Sick Children (HSC) and the University of Toronto. All participants in this study will be required to provide written informed consent. The consent form will include a statement that participation is voluntary and that participants can withdraw from the study at any time. Participants will be sent a copy of the consent form and a one-page coping agreement *via* email prior to the start of the first session so they can review it. At the start of the first program session, 30 min will be set aside for study administration (including answering any additional questions the participants have), reviewing and signing the consent form, filling out the one-page coping agreement and filling in a social demographic survey. Confidentiality and data management have been addressed to fulfill the requirements of the research ethics boards that reviewed this study.

## Outcome Measures

### Primary Outcome Measure: Semi-structured Post-intervention Interviews

The primary data for this study will be generated through qualitative semi-structured post-program interviews. All participants will be invited to participate in a post-program interview. The interviews will be conducted with participants within 4 weeks of the final session of the 5-week AMP-MP program. Semi-structured interviews have been chosen as they allow for a clear sequence of themes and questions, while also accounting for the necessity to adapt the order and wording of questions throughout the interview process based on participants' specific responses (60). Interviews will be conducted either in-person, or over a secured video conferencing platform. All interviews will be audio recorded and transcribed.

#### Development and Iteration of Interview Guide

The study's interview guide was developed with the aim to generate richly detailed data that will get at the “essence or inner core” of participants' experience. High-quality interview guides keep in mind both the bigger picture/arc of the interview (e.g., the overarching sequence of questions) along with the more granular elements (e.g., individual probes) (61). Therefore, the development of this study's interview guide aims to foster (1) a natural conversational flow across the interview and (2) simultaneously delve deeply into specific components of each participants' unique experience.

### Secondary Outcome Measure: Participant Observation

Participant observation will be conducted to better understand the lived experience of participants within the context of the program, rather than only from participants' post-program interviews (62). Detailed field notes will be generated by the participant observer (EW), who will be present during all program sessions for the three study groups. The participant observation field notes will be used to develop documents summarizing the characteristics of each group to further inform data analysis and interpretation.

## Reflexivity and Positionality

Reflexivity is central to the work of qualitative research. The researcher's position is influenced and shaped by a multiplicity of factors that include the institutional, cultural, historical and interactional contexts (63–66). In the case of this particular study, the lead researcher's long-standing experience as a mindfulness practitioner provides a unique perspective through which to generate and interpret the data. While this lens provides knowledge useful to deploy in relation to the aims of the study, it also has embedded within it assumptions that could impact data analysis (64, 67, 68). Therefore, analytic strategies (e.g., reading for contradictions and reflexivity as an analytic tool) will be applied during analysis and interpretation (69–71).

## Research Quality: Approaches to Developing High-quality Rigorous Qualitative Research

This section discusses the specific processes and strategies that will be applied across the study to develop rigor. Qualitative methodologists have argued the need for qualitative studies to have their own approaches to rigor, as there is a concern that a proceduralist orientation to rigor can over-simplify the complex dynamic nature of qualitative inquiry (72, 73). Therefore, this study has identified and will implement the following approaches and strategies to developing rigor in congruence with the ontology and epistemology of the paradigm being used to guide this study, which is the interpretive paradigm.

### Theoretical Perspectives

The application of theory throughout the research process is central to the development of rigor and congruence across a qualitative study. The interpretative paradigm will be mobilized across all phases of this research study. The interpretive paradigm is well suited to this study's research aims as it seeks to *understand* human behavior, rather than *explain* it (42). The Mechanisms of Mindfulness Theory (MMT) (74) was selected as the middle range theory for this research study. A middle range theory acts as a link between a more macro (abstract) theoretical perspective and a more granular perspective about the behavior of individuals in everyday settings (42, 66). MMT expands upon established theories of attention with additional theory that illuminate skillsets particular to the phenomenon of mindfulness. MMT has three main components that aim to describe the underlying mechanisms that are activated through the development of mindfulness. These mechanisms are seen to undergird the benefits described in the literature (e.g., reduced anxiety/stress and increased empathy/wellbeing). The three components include: (1) The IAA model made up of three axioms (Intention, Attention, and Attitude); (2) An overarching (meta) mechanism of Reperceiving (significant shift in perspective); and (3) Four additional mechanisms: (1) Self-Regulation and Self-Management; (2) Values Clarification; (3) Cognitive, Emotional; and (4) Behavioral Flexibility; Exposure (74). These three components are described in more detail within in the Supplementary Materials labeled “Mechanisms of Mindfulness Theory Summary”.

### Approach to Transcription

Three key strategies will be used to produce transcripts that reflect the original nature of the verbal accounts and minimize potential transcription errors. These included: (1) Providing detailed transcription guidelines to the transcriptionist (see Supplementary Materials); (2) Reviewing the first transcription against the audio recording and providing further guidelines prior to the rest of transcription; and (3) Reviewing the transcriptions against the original audio recordings before proceeding with textual analysis.

### Member Reflections

*Member reflection* will be applied as part of the data analysis and write up process to enhance the quality of this study through the process of seeking questions, critiques, feedback and affirmation from participants (71). *Member reflection* will be collected by the participant observer during focus group sessions which will be conducted 16-months after the initial study.

### Thematic Analysis

Thematic Analysis (TA) will be applied to analyze and interpret the data. The six steps of TA outlined by Braun and Clarke provide a robust framework that allows for a clear and systematic approach (75, 76). The six steps of TA include: (1) Familiarization with the data, (2) Generating initial codes, (3) Searching for themes, (4) Reviewing themes, (5) Defining and naming themes, and (6) Producing the report.

Within each step of TA there are a multiplicity of analytic choices that reflect the interplay of method and theory. A primarily inductive approach will be applied, with the themes remaining strongly linked to the data, or “data-driven”, rather than fitting into a preconceived coding format based on a priori assumptions. NVivo software will be used to manage the coding process but will not be seen as a tool for analysis or interpretation.

## Discussion

### Strengths and Limitations

This study has several noteworthy features that might be seen as limitations in some contexts. The sample will be based on physicians who choose to respond to the recruitment efforts, therefore self-selecting to participate in the mindfulness program. Self-selection can be viewed as a practical approach for delivering MBIs in healthcare settings, as an individual's *intention* to engage is seen as one of the underlying axioms that activate the mechanisms that undergird the development of mindfulness (74).

While a purposive sampling approach was selected to enhance the specificity of the sample and increase the sample's information power, there is also an element of convenience sampling as the sample will be based on those who respond to the recruitment efforts. The purposive sampling approach (e.g., physicians with greater years of medical practice and fewer years of mindfulness experience) helps focus the study on the population of physicians. Therefore, it will not be known how medical students or other healthcare personnel (HCPs) would experience the Applied Mindfulness program.

The two program facilitators have been selected based on having an ideal set of credentials to deliver the Applied Mindfulness Program. While this will support the successful delivery of the program, it will limit the understanding of whether the program could be effectively taught and scaled into other contexts that may not have access to facilitators with the same level of expertise.

The use of semi-structured interviews as the primary data outcome may be considered by some as a limitation. This data set can be seen as best suited to answer the aims of this study's research question and also address gaps in the current literature regarding the acceptability and pragmatic application of mindfulness in physician's daily lives.

### Implications for Policy, Research, and Practice

This study seeks to add pragmatic resources to the field through a prospective qualitative study that brings together the phenomena of physician wellbeing and mindfulness. Very few empirical studies have brought together these phenomena, with almost none using qualitative methods. To our knowledge, at the time of this publication, no research studies have used the teachings of Thich Nhat Hanh as the explicit basis for an MBI. Therefore, our innovative study design aims to answer calls from both the literature and international medical associations to determine the acceptability and feasibility of an Applied Mindfulness intervention for the improvement of physician wellness.

Our study design takes into account how this modality can be delivered in an accessible way for both the participants (e.g., delivering the program onsite, length of program/sessions, allowing participants to select the time/day that suits their schedules etc.) and the institutions that may wish to implement it (e.g., low-cost, approaches to curating a mindful space onsite etc.). As such, our results also aim to provide recommendations on best approaches for implementing this type of training and research within hospital and healthcare settings.

Burnout amongst physicians has been recognized across the literature as highly prevalent, and increasingly so in the context of major public health crises such as the COVID-19 pandemic. Therefore, if results from this study show our Applied Mindfulness intervention to be an acceptable and feasible modality, it could support trainings in Applied Mindfulness being incorporated into hospital settings as an effective means to improve individual physicians' wellbeing. In addition to this, findings in this study will address the question of whether training physicians in Applied Mindfulness can simultaneously benefit their patients and the wider healthcare system.

## Ethics Statement

The studies involving human participants were reviewed and approved by The Hospital for Sick Children (HSC) and the University of Toronto. The patients/participants provided their written informed consent to participate in this study.

## Author Contributions

EW was the primary contributor to the conception, design of the study, and wrote the first draft of the manuscript. NC contributed to the conception, design of the study, and provided critical revisions and checked the manuscript for important intellectual content. All authors approved of the final version of the manuscript as submitted.

## Funding

This study was funded by a Supervisor's Research Grant through The Hospital for Sick Children and the Institute of Medical Sciences, University of Toronto (EW). NC is supported by a Junior 1 Career Development Award from the Fonds de Recherche du Québec—Santé.

## Conflict of Interest

The authors declare that the research was conducted in the absence of any commercial or financial relationships that could be construed as a potential conflict of interest.

## Publisher's Note

All claims expressed in this article are solely those of the authors and do not necessarily represent those of their affiliated organizations, or those of the publisher, the editors and the reviewers. Any product that may be evaluated in this article, or claim that may be made by its manufacturer, is not guaranteed or endorsed by the publisher.
