# Supplementary material for: Applied Mindfulness for Physician Wellbeing: A Prospective Qualitative Study Protocol
Source: Front Public Health. 2022 Feb 11;10:807792. doi: 10.3389/fpubh.2022.807792 (PMC8873143; doi:10.3389/fpubh.2022.807792)
Supplement: Supplementary file 1 [file Table_1.pdf]

Supplementary Material

Transcription guidelines

Adapted from Poland, B. (1995). Transcription quality as an aspect of rigor in qualitative research. *Qualitative Inquiry, 1*(3), 290-310.

Labeling transcription files

- In the header of each transcription file please include:
    - Study name “Applying Mindfulness to Physician Wellbeing”
    - Interview date (indicated in the label of each audio file)
    - Participant number (indicated in the label of each audio file)
- Transcription file names: please label each transcription file by…
    1. Interview date, e.g., May 5-19 (indicated in the label of each audio file)
    2. Participant # (indicated in the label of each audio file)
    - E.g., May 5-19 #56

General

- Before question #1, ask if participants would like to start with a mindfulness practice. Please treat this as the first question and include this question and their answer in the transcript.
- Include timecode throughout the transcripts roughly every ten minutes or when a new question is asked, e.g., 10:48 for ten minutes and forty-eight seconds into an interview.

Transcription Symbols
I = interviewer
P = participant

**Symbol**	**Description**	**Example**
[ ]	Square brackets indicate a point at which the current speaker's talk is overlapped by another speaker	P: Well I think [yeah] that I really
(.) (..) (…)	A dot in parentheses indicates a shorter pause or silence, something long enough to be of note but under .10 seconds; one dot for a short pause, two or three dots the longer the pause	I can’t remember (.) maybe last week
_______	Underscoring indicates a strong emphasis on a certain word	That’s <u>amazing</u> or I found that <u>stressful</u>

Transcription guidelines

Adapted from Poland, B. (1995). Transcription quality as an aspect of rigor in qualitative research. *Qualitative Inquiry, 1*(3), 290-310.

ALL CAPS	Capitals, except at the beginning of a sentence, indicate especially loud sounds relative to rest of talking	WOW super interesting
.hhhhh	A row of h's prefixed by a dot indicates an in-breath; without a dot, an out-breath. The length of the row of h's indicates the length of the in- or out breath	When I meditate, I take a deep breath .hhhhh
( )	Empty parentheses indicate the transcriber's inability to hear what was said	Then I thought ( ) and afterwards I went outside
(word)	Parenthesized words are possible hearings but not certain	Then I thought (it's ok) and afterwards I went outside
(( ))	Double parentheses contain descriptions rather than transcription -> e.g. laugh, cough, sneeze, sigh	That is so funny ((laughs)) I never thought of that
?	Indicates speaker's intonation	What do you think?
" "	Quotations are used to indicate if the speaker is parodying what someone else said or an inner voice in their heads	My sister always says "don't get so stressed" but I never listen
