# Supplementary material for: Applied Mindfulness for Physician Wellbeing: A Prospective Qualitative Study Protocol
Source: Front Public Health. 2022 Feb 11;10:807792. doi: 10.3389/fpubh.2022.807792 (PMC8873143; doi:10.3389/fpubh.2022.807792)
Supplement: Supplementary file 2 [file Table_2.pdf]

frontiers

Supplementary Material

<table>
<tr><td colspan="2">Mechanisms of Mindfulness Theory Summary

Adapted from Shapiro, S., Carlson, L., Astin, J., & Freedman, B. (2006). Mechanisms of mindfulness. J Clin Psychol, 62(3), 373-386. doi:10.1002/jclp.20237</td></tr>
<tr><td colspan="2">1: Foundational Mechanisms - IAA Model
They posit three axioms of mindfulness that work in concert (non-linearly) to produce “mindfulness”</td></tr>
<tr><td>Axiom 1: Intention</td><td>The dynamic and evolving personal vision behind why one chooses to practice mindfulness, often moving along a continuum from self-regulation, to self-exploration, to self-liberation.</td></tr>
<tr><td>Axiom 2: Attention</td><td>Self-regulation of attention to observe one’s moment-to-moment, internal and external experiences.</td></tr>
<tr><td>Axiom 3: Attitude</td><td>The qualities of attention one brings to how they are attending, examples of mindfulness attitudes include patience, openness, kindness, compassion and non-striving.</td></tr>
<tr><td colspan="2">2: Meta Mechanism - Reperceiving
The activation of the three axioms (IAA model above) leads to a significant shift in perspective that the authors see as a meta mechanism and label with the term “reperceiving”</td></tr>
<tr><td>Reperceiving</td><td>• Involves a fundamental shift in perspective
• Shifting perspective like this is a natural part of the cognitive developmental process, mindfulness continues or accelerates this process
• Reperceiving facilitates greater distance in terms of clarity, but does not translate to disconnection our disassociation
• Allows deep experiences of events without clinging to them
• Allows for greater richness, texture and depth of experiences, not apathy or indifference</td></tr>
<tr><td colspan="2">3: Additional Mechanisms
Four additional mechanisms are identified as potential products or results of activating the foundational mechanisms of IAA and the Meta Mechanisms of reperceiving</td></tr>
</table>

**Mechanisms of Mindfulness Theory Summary**<br><br>*Adapted from Shapiro, S., Carlson, L., Astin, J., & Freedman, B. (2006). Mechanisms of mindfulness. J Clin Psychol, 62(3), 373-386. doi:10.1002/jclp.20237*	
Self-Regulation & Self-Management	• A system's ability to maintain stability and adaptability in the face of change<br>• Self-regulation is based on feedback loops that can be enhanced by intention and attention to create greater health
Values Clarification	• Enhances the ability to recognize what is meaningful and what is truly valued (with awareness of how these may be conditioned by family, culture and society)<br>• Helps support identifying and choosing behaviors that are congruent with current needs, interests and values
Cognitive, Emotional & Behavioral Flexibility	• Greater clarity analyzing a situation's impact on our own internal reactions which allows for...<br>‣Adaptive, flexible responding to the environment<br>‣Responding in less conditioned automatic ways
Exposure	• Capacity to observe and experience (be exposed to) strong emotions with greater objectivity and less reactivity<br>• Counters the habit of avoiding or denying difficulty and therefore increases exposure to these states<br>• Through exposure, difficulty can become less overwhelming or less fear inducing
